# G3 PhyloChip Analysis Confirms the Promise of Plant-Based Culture Media for Unlocking the Composition and Diversity of the Maize Root Microbiome and for Recovering Unculturable Candidate Divisions/Phyla

**DOI:** 10.1264/jsme2.ME18023

**Published:** 2018-09-29

**Authors:** Mohamed S. Sarhan, Sascha Patz, Mervat A. Hamza, Hanan H. Youssef, Elhussein F. Mourad, Mohamed Fayez, Brian Murphy, Silke Ruppel, Nabil A. Hegazi

**Affiliations:** 1 Environmental Studies and Research Unit (ESRU), Department of Microbiology, Faculty of Agriculture, Cairo University Giza, 12613 Egypt; 2 Leibniz Institute of Vegetable and Ornamental Crops Großbeeren/Erfurt e.V. (IGZ) Großbeeren, 14979 Germany; 3 Department of Botany, School of Natural Sciences, Trinity College Dublin Dublin 2 Ireland; 4 Algorithms in Bioinformatics, Center for Bioinformatics, University of Tübingen Tübingen, 72076 Germany

**Keywords:** Plant microbiome, plant-based culture media, unculturable bacteria, *Candidatus* Phytoplasma, candidate phyla, divisions

## Abstract

The rapid development of high-throughput techniques and expansion of bacterial databases have accelerated efforts to bring plant microbiomes into cultivation. We introduced plant-only-based culture media as a successful candidate to mimic the nutritional matrices of plant roots. We herein employed a G3 PhyloChip microarray to meticulously characterize the culture-dependent and -independent bacterial communities of the maize root compartments, the endo- and ecto-rhizospheres. An emphasis was placed on the preference of the growth of unculturable candidate divisions/phyla on plant-only-based culture media over standard culture media (nutrient agar). A total of 1,818 different operational taxonomic units (OTUs) were resolved representing 67 bacterial phyla. Plant-only-based culture media displayed particular affinity towards recovering endophytic over ectophytic rhizobacteria. This was shown by the slightly higher recovery of CFUs for endophytes on plant-only-based culture media (26%) than on standard culture media (10%) as well as the higher taxa richness and numbers of exclusive families of unculturable divisions/phyla. Out of 30 bacterial phyla (comprising >95% of the whole population), 13 were of a significantly higher incidence on plant-only-based culture media, 6 phyla of which were not-yet-cultured (*Atribacteria*, OP9; *Dependentiae*, TM6; *Latescibacteria*, WS3; *Marinimicrobia*, SAR406; *Omnitrophica*, OP3; BRC1). Furthermore, plant-only-based culture media significantly enriched less abundant and/or hard-to-culture bacterial phyla (*Acidobacteria*, *Gemmatimonadetes*, and *Tenericutes*). These results present conclusive evidence of the ability of plant-only-based culture media to bring the plant-fed *in situ* microbiome into the status of plant-fed *in vitro* cultures, and to widen the scope of cultivation of heretofore-unculturable bacterial divisions/phyla.

Plant roots are very active integrated ecosystems that harbor massive numbers of bacterial species comprising the root microbiome. These bacteria essentially contribute to plant nutrition and health (3, 38, 39, 50). Therefore, the study of the root microbiome is of great importance for various biotechnological applications, *e.g.* plant-growth promotion, biological control, and/or the production of bioactive compounds. Unfortunately, most of these plant-inhabiting bacterial species (>90%) are non-culturable and may play principal and often unique roles in root ecosystem functions (3, 25, 58). To date, the highly diverse bacterial community has been partially examined using cultivation-based methods (49), and the use of culture-independent methods has revealed unknown members of plant microbiomes, resulting in high-throughput data related to the unculturable bacterial components of the plant microbiome (2).

The recent development of metagenomics led to a series of studies that performed functional analyses on entirely uncultured bacterial groups (5, 40, 46, 53, 60, 63). However, the cultivation of these bacteria is essential for functional understanding and further microbiological and biotechnological applications. Therefore, efforts are continually exerted towards bringing these not-yet-cultured organisms into axenic cultures by developing new growth media with various substrate compositions and modifying existing culture media and cultivation methods. These modifications include the addition of nutritional supplements and dilution of nutrient concentrations, together with the prolongation of incubation periods (23, 51). The imitation of natural environments within culture media represents another major approach (6, 17, 19). We recently proposed the usage of plant materials as the sole source of nutrients in culture media to investigate a wider range of the plant microbiome (43, 51, 62).

The majority of bacterial clades in the newly introduced tree of life (26) have only been identified through analyses of bulk environmental samples and metagenomics, and the number of environmental 16S rRNA gene sequences has greatly surpassed that of cultured microorganisms; therefore, the taxonomic assignment of these sequences is lagging (11, 61). Consequently, the precise number of bacterial phyla that may be recoverable currently remains unknown. According to the Greengenes database (greengenes.lbl.gov), estimates of bacterial phylum numbers currently range between 31 and 88 depending on which of the five different taxonomy systems are used (Pace, Hugenholtz, Ludwig, RDP, or NCBI). The main reason behind this discrepancy is the inability to culture a number of bacterial phyla in order to accommodate them within a systematic classification. Therefore, continuous efforts for the *in vitro* culturing of representatives of these candidate phyla/divisions remain a valid approach to achieve a unified bacterial taxonomy.

In the present study, we analyzed the microbiome of maize (*Zea maize* L.) root compartments (endo- and ecto-rhizospheres) to compare culture-dependent versus culture independent bacterial communities, in terms of CFU counts versus total bacterial qPCR counts, and the resulting comparative taxonomic characterization of these communities. We employed G3 PhyloChip microarrays to investigate the taxonomic affiliation of culture-dependent bacterial communities, developed on new plant-only-based culture media versus standard artificial nutrient agar, in relation to root culture-independent bacterial communities. Our primary objective was to highlight the effects of culture medium on the incidence and abundance of unculturable candidate bacterial phyla/divisions. The G3 PhyloChip used in the present study accommodates 1.1 million DNA probes with the ability to categorize all known bacteria and archaeal operational taxonomic units (OTUs) into more than 50,000 taxa using 59,959 clusters of 17 nucleotides as probes representing 147 phyla, 1,123 classes, 1,219 orders, and 1,464 families, for a total of 27,938 OTUs (20).

## Materials and Methods

### Plant sampling

Samples representing the vegetative parts and root system of fully mature maize plants (*Zea mays* L.) were obtained from the experimental fields of the Faculty of Agriculture, Cairo University, Giza, Egypt (30°01′03.7″N 31°12′19.1″E). They were transferred to the laboratory in plastic bags, and used directly for culture-dependent and culture-independent analyses.

### Culture-independent quantification of total bacterial numbers (qPCR)

To quantify total bacterial numbers in endo- and ectorhizosphere samples, 16S rRNA gene copy numbers were measured using the universal primers 519f and 907r, which are specific for the bacterial domain (33), and a real-time PCR analysis with SYBR^™^ Green I as an intercalating dye. Quantification and the cycling program were performed according to methodologies described in detail by Sarhan *et al.* (51). Data calculations were conducted using CFX^™^ optical systems software and a calibration curve established by a ten-fold dilution (range between 10^9^ to 1 copy μL^−1^) of specific *Escherichia coli* PCR products. The quality of the quantification method was verified using a melting profile, giving one specific melting peak at 87°C, and running an agarose gel detecting one single band at a size of 407 bp. Bacterial cell numbers were calculated indirectly by assuming that the average rRNA operon copy number cell^−1^ was 3.6 (31, 52).

### Culture-dependent analysis

Regarding the ectorhizosphere (outer root compartment representing the rhizoplane together with closely adhered rhizosphere soil), plant roots were shaken vigorously and a known weight of roots was transferred to the half-strength basal salts of CCM liquid medium as a diluent (22). Further serial dilutions were prepared (10^−2^–10^−7^). Regarding the endorhizosphere (inner root compartment), plant roots were carefully washed with tap water, surface-sterilized with 95% ethanol for 30 s followed by 3% sodium hypochlorite for 30 min, then thoroughly washed five times with sterile distilled water. Five grams of surface-sterilized roots was blended for 5 min in a Waring blender using 45 mL of the half-strength basal salts of CCM liquid medium as a diluent, and further serial dilutions were prepared. Aliquots of 200 μL of suitable dilutions representing both root compartments (ecto- and endorhizosphere samples) were surface-inoculated on agar plates of the culture media being tested. Agar plates were incubated at 28°C for 2–10 d, and colony-forming units (CFUs) were periodically examined and counted.

### Culture media

*Plant-only-based culture media* (51): We used clover plants (*Trifolium alexandrinum* L.) because of its diverse nutrient composition of C and N compounds, macro- and microelements, and amino acids and vitamins (51). This nutrient composition supports its ability to satisfy the growth of rhizobacteria associated with various host plants (43, 51). Plant teabag culture media were prepared according to Sarhan *et al.* (51). Shoots of fully-grown clover (*T. alexandrinum* L.) were dehydrated in the sun for 24 h and then oven dried at 70°C for 24 h. Dehydrated plant materials were mechanically ground to pass through a 2.0-mm sieve in order to obtain fine dehydrated powder. Teabags were prepared by packing two grams of the dehydrated powder into each bag and sealing by stapling. Two teabags (each containing 2 g) were added to 1 L of distilled water in order to obtain the liquid plant infusion. Solid culture media were prepared by adding agar (2%, w/v), adjusting pH to 7.0, and then autoclaving at 121°C for 20 min. The teabags were left in the culture media during autoclaving for further plant extraction. Media were tested to ensure sterility before use.

Nutrient agar: Standard nutrient agar was used and comprised the following (g L^−1^): beef extract, 3.0; peptone, 5.0; glucose 1.0; yeast extract 0.5.

Agar culture media were prepared by adding agar (2%, w/v) and autoclaving at 121°C for 20 min. All culture media were proved to ensure sterility before use.

N-deficient combined carbon-source medium (CCM) basal salts by Hegazi *et al.* (22): This formulation comprised the following (g L^−1^): K_2_HPO_4_, 0.4; KH_2_PO_4_, 0.6; MgSO_4_, 0.2; NaCl, 0.1; MnSO_4_, 0.01; KOH, 1.5; CaCl_2_, 0.02; FeCl_3_, 0.015; Na_2_ MoO_4_, 0.002; in addition to CuSO_4_, 0.08 mg L^−1^; ZnSO_4_, 0.25 mg L^−1^.

### Sample preparation for PhyloChip hybridization

To characterize the composition of culture-dependent and -independent microbial communities, three representative samples of each root compartment (endo- and ectorhizospheres) were included: 1) the cultured community of the tested plant-based culture media; 2) the cultured community grown on standard nutrient agar; and 3) the native microbial community of maize roots without enrichment. Three replicates of each sample were included in analyses.

### Harvest of microbial colonies and DNA extraction

Regarding the DNA extraction of culture-dependent communities (samples 1 and 2), all CFUs developed on representative 10-d-incubated agar plates of the tested culture media were washed using 0.05 M NaCl solution, and collected by centrifugation at 9,500×*g* for 10 min. DNA was extracted from collected CFU pellets using the QIAGEN DNeasy plant mini kit (QIAGEN, Hilden, Germany) according to the manufacturer’s instructions. In culture-independent analyses (sample 3), total DNA was extracted from 5-mL aliquots of the original root suspensions of ecto- and endorhizosphere samples (three replicates each) that were previously prepared to make serial dilutions for the culture-dependent analysis. DNA concentrations were measured at 260 nm, DNA quality was checked photometrically by a A_260_/A_280_ ratio calculation to be greater than 1.9, and the A_320_ measurement was nearly 0 using NanoDrop 2000 UV-Vis Spectrophotometer (Thermo Fisher Scientific, Waltham, MA, USA).

### Amplification of 16S rRNA gene and PhyloChip hybridization

Using the DNA from plate harvest and original root suspensions, bacterial 16S rRNA genes were amplified using the degenerate forward primer 27f: AGRGTTTGATCMTGGCTCAG, and the non-degenerate reverse primer 1492r: GGTTACCTTGTTACGACTT. Thirty-five cycles of amplification were performed, and the amplified products were fragmented, biotin labeled, and hybridized to the PhyloChip^™^ Array, version G3 following the procedures described by Hazen *et al.* 2010 (20). PhyloChip arrays were washed, stained, and scanned using a GeneArray^®^ scanner (Affymetrix, Santa Clara, CA, USA) (Fig. S1). Each scan was captured using standard Affymetrix software (GeneChip^®^ Microarray Analysis Suite). Samples were processed in a Good Laboratory Practices (GLP) compliant service laboratory running Quality Management Systems for sample and data tracking. The laboratory (Second Genome’s service laboratory, South San Francisco, CA, USA) implements detailed standard operating procedures (SOPs), equipment and process validation, training, audits and document control measures. Quality control (QC) and quality assurance (QA) metrics were maintained for all sample handling, processing, and storage procedures. The detailed PhyloChip protocol is described in Methods S1.

### Data summarization and statistical analysis

After taxa were identified for inclusion in the analysis, the values used for each taxa-sample intersection were populated in two distinct manners; Hybridization Scores (HybScores, Fig. S1) used directly to denote abundance (Data S4), and incidence scores to denote presence/absence (Data S5). Detailed information on data summarization are provided in Methods S1.

Second Genome’s PhyloChip processing software was used to inter-compare all sample profiles in a pair-wise manner, and UniFrac distances (36) were utilized to assess the metric distance between tested communities. In Weighted UniFrac, OTU abundance was additionally considered, whereas UniFrac was used for presence/absence data. A two-dimensional ordination Principal Coordinates Analysis (PCoA) and hierarchical clustering maps of samples in the form of dendrograms were created to graphically summarize inter-sample relationships. In whole microbiome significance testing, the Adonis test was utilized to identify significant differences among weighted and unweighted data.

We used the following R-project packages (https://cran.r-project.org/): “pvclust” was used to calculate bootstrap probability percentages, “VennDiagram” was used to construct Venn diagrams that represent the overlapping of taxa using incidence data based on the family level, and the “gplots-heatmap.2” function was used to construct heatmaps of the abundance of phyla and OTUs.

The Student’s *t*-test was applied across tested samples, and heatmaps were generally used to display OTUs with significant differences (*P*≤0.05) in abundance. Among the 1160 OTUs, representing 206 families, we constructed the heatmap shown in Fig. 3B to compare the abundance of 218 OTUs with significant differences among plant-based culture media and nutrient agar. The circular tree shown in Fig. S5 was constructed using the reference number of each bacterial group in PhyloT (http://phylot.biobyte.de/) based on NCBI taxonomy. Taxonomy labels and abundance data are rendered in iTOL (34) (http://itol.embl.de/). The rings around the tree comprise a heatmap: blue indicates a more abundant OTU in that sample than in the mean of the baseline samples, while yellow represents a less abundant OTU, and color saturation shows the degree of the difference from the mean value of baseline samples.

## Results

### Culture-dependent and -independent quantification of the maize root microbiome

**Fig. 1:**
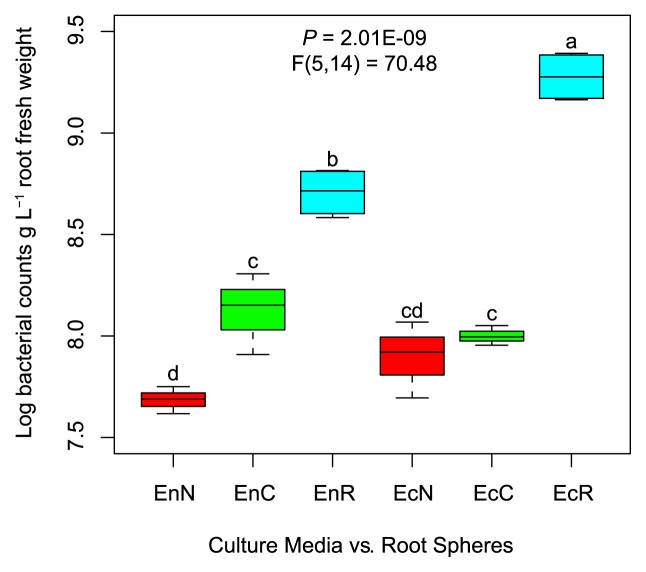
Culture-dependent and -independent recovery of rhizobacteria associated with maize root compartments. Log numbers of CFUs of culturable rhizobacteria in the endorhizosphere (En) and ectorhizosphere (Ec) as developed on nutrient agar (EnN, EcN), and plant-based culture medium (EnC, EcC). Total numbers of rhizobacteria measured by qPCR in maize root compartments of the endorhizosphere (EnR) and ectorhizosphere (EcR). Significant differences are indicated by different letters at *P*≤0.05 (Tukey’s HSD).

We compared culture-dependent (plant-based culture media and nutrient agar) and -independent (qPCR) bacterial communities of the inner (endorhizosphere) and outer (ectorhizosphere) compartments of maize roots. Culture-independent bacterial cell numbers were significantly lower in the endorhizosphere (log 8.7±0.061 bacterial cells g^−1^ root) than in the ectorhizosphere (log 9.29±0.06 bacterial cells g^−1^ root) (Fig. 1). Culture-dependent populations generally represented <30% of culture-independent total bacterial numbers in the endorhizosphere, with significantly higher values for plant-based culture media (26%) than for nutrient agar (10%) (Fig. 1). While culturable populations in the ectorhizosphere represented only 4–5% of culture-independent bacterial numbers, no significant differences were attributed to the tested culture media. Plant-based culture media revealed the prominent development of microcolonies (μ-colonies, <1 mm diameter discriminated with 40× magnification, Fig. S2).

### G3 PhyloChip-based culture-dependent and -independent prokaryotic community analysis

**Fig. 2:**
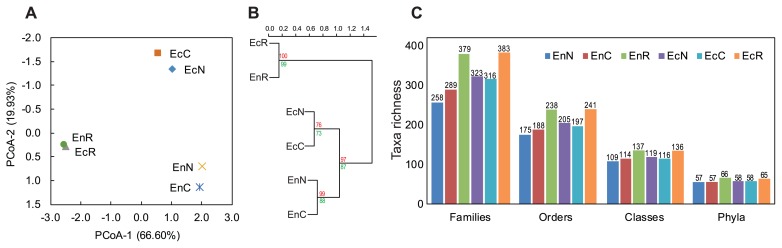
Analysis of culture-dependent (CFUs) and culture-independent (maize roots) bacterial and archaeal community compositions of maize root compartments based on G3-PhyloChip technology. A, Principal Co-ordinates Analysis (PCoA); B, Hierarchical clustering, bootstrap probabilities (%) are indicated in green and approximately unbiased *p*-values are shown in red; C, Bacterial richness at different taxonomic levels (phyla, classes, orders, families). En, Endorhizosphere; Ec, ectorhizosphere; R, culture-independent root sphere; C, plant-based culture media; N, nutrient agar culture media.

G3 PhyloChip microarrays were used to analyze the culture-dependent (CFU-harvest of representative agar plates) and culture-independent (original suspensions of the tested root compartments) bacterial and archaeal communities of the endorhizosphere and ectorhizosphere of the maize plant. A total of 1,818 different OTUs, comprising bacteria and archaea, were detected in all of the samples analyzed (Fig. S3). Bacteria were represented by 1,769 OTUs (67 phyla, 146 classes, 260 orders, 423 families, 704 genera, and 987 species), and Archaea corresponded to 49 OTUs (2 phyla, 8 classes, 12 orders, 17 families, 26 genera, and 32 species) (Fig. S3). Considering the presence/absence (incidence, unweighted data) and abundance (weighted data) of all 1,818 OTUs, significant differences were attributed to the root compartments, namely, the endorhizosphere versus ectorhizosphere, and analysis methods, that is, culture-dependent versus culture-independent communities (Table S1). A Principal Co-ordinates Analysis (PCoA) and hierarchical clustering revealed a clear separation between culture-dependent (samples representing both culture media) and -independent communities (samples representing plant roots), with subsequent separations among culture-dependent communities according to the root compartments, the endorhizosphere and ectorhizosphere (Fig. 2A and B).

Our results indicated that the richness of bacterial communities was slightly higher in the ectorhizosphere than in the endorhizosphere, regardless of the culture-dependent or the -independent communities. Disparities among the culture media tested were not detected at the phylum level, but started to appear at lower taxonomic levels to orders and families. These disparities were strongly distinguished in the endorhizosphere, particularly with plant-based culture media (Fig. 2C).

### Commonalities and differences among culture-dependent and -independent bacterial communities

**Fig. 3:**
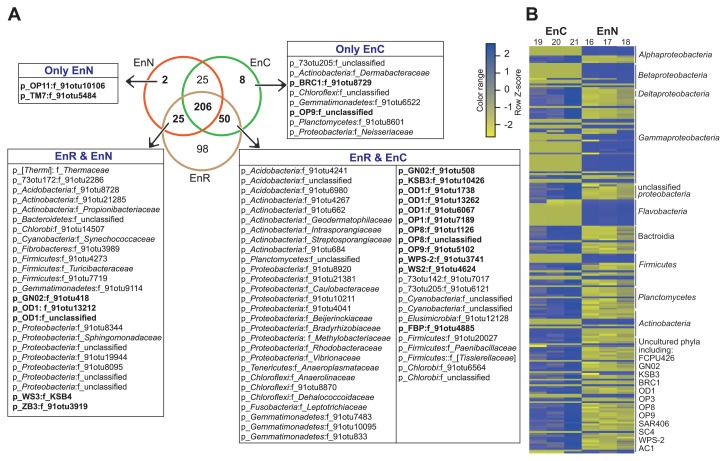
Overlapping of culture-dependent (on plant-based culture medium and nutrient agar) and -independent bacterial communities of the maize root endorhizosphere. A, Venn diagram at the family level for bacterial communities displaying unique and overlapping families; families exclusively grown on only one of our tested media are shown in the linked boxes, and not-yet-cultured candidate divisions are marked in bold; B, Heatmap of weighted abundance of the OTUs that displayed significant differences among the 206 families commonly grown on both culture media (EnN, nutrient agar; EnC, plant-based culture medium, three replicates shown for each medium). Please refer to Data S1 for detailed information.

The results obtained indicated limitations in the PhyloChip method for securing continuous taxonomic annotation beyond families down to species level. Therefore, we restricted our analysis to the family level, and used richness (the number of families) to analyze the overlap between culture-dependent and culture-independent bacterial communities, *i.e.* culture media versus root compartments. Regardless of the method used, 414 and 418 different bacterial families were detected in all of the tested samples representing the endo- and ectorhizospheres, respectively (Fig. 3A, Fig. S4, and Data S1).

Among the 414 families detected in the endorhizosphere, 98 (representing 23.7% of all families detected) were exclusively detected by the culture-independent method. On the other hand, 35 families were only resolved by the culture-dependent method; of these, 25 were common among plant-based culture media and nutrient agar, 8 were unique for plant-based culture media (2 belonged to the uncultured phyla/divisions BRC1 and *Atribacteria*, OP9), and 2 were unique for nutrient agar (belonged to *Microgenomates*, OP11 and *Saccharibacteria*, TM7). Plant-based culture media shared 50 families with culture-independent communities that were unable to grow on nutrient agar. Twelve of these families belonged to the unculturable candidate phyla/divisions of FBP, *Gracilibacteria* (GN02), *Modulibacteria* (KSB3), *Parcubacteria* (OD1), *Acetothermia* (OP1), *Aminicenantes* (OP8), *Atribacteria* (OP9), WPS-2, and WS2. This is in addition to the number of rarely and/or difficult-to-isolate families belonging to the phyla *Proteobacteria* (*Caulobacteraceae*, *Beijerinckiaceae*, *Bradyrhizobiaceae*, *Methylobacteriaceae*, *Rhodobacteraceae*, and *Vibrionaceae*), *Actinobacteria* (*Geodermatophilaceae*, *Intrasporangiaceae*, and *Streptosporangiaceae*), *Chloroflexi* (*Anaerolinaceae* and *Dehalococcoidaceae*), *Firmicutes* (*Paenibacillaceae* and *Tissierellaceae*), *Fusobacteria* (*Leptotrichiaceae*), and *Tenericutes* (*Anaeroplasmataceae*) (Fig. 3A). On the other hand, nutrient agar shared lower numbers of families, only 25, with culture-independent communities, which were not detectable on plant-based culture medium. Five of these families belonged to the unculturable candidate phyla/divisions *Gracilibacteria* (GN02), *Parcubacteria* (OD1), *Latescibacteria* (WS3), and ZB3 (Fig. 3A). Two hundred and six families were commonly detected by both methods, and cultured on both culture media. Since we are interested in the effects of culture media, we used the abundance of OTUs representing these families to compare the enrichment potential of both tested culture media and their suitability for recovering specific bacterial groups. These 206 families were represented by 1,160 OTUs, 218 of which displayed significant differences among plant-based culture media and nutrient agar (Fig. 3B). We found that the OTUs belonging to *Betaproteobacteria*, *Gammaproteobacteria*, and *Flavobacteria* were particularly enriched on nutrient agar. In contrast, plant-based culture media preferentially recovered the taxa of *Deltaproteobacteria*, *Bacteroidia*, *Actinobacteria*, *Tenericutes*, *Fibrobacteres*, *Chlamydiae*, *Cyanobacteria*, *Elusimicrobia*, *Gemmatimonadetes*, and *Nitrospirae*, as well as a number of uncultured candidate phyla/divisions (FCPU426, *Gracilibacteria* [GN02], *Modulibacteria* [KSB3], BRC1, *Parcubacteria* [OD1] *Aminicenantes* [OP8], *Atribacteria* [OP9], *Marinimicrobia* [SAR406], SC4, and WPS-2) (Fig. 3B and Data S1).

When we examined overlaps in the ectorhizosphere, 61 families were detected by culture-independent methods only. However, only 35 families were resolved by culture-dependent methods; 5 on plant-based culture media, 7 on nutrient agar, and 23 on both. In comparisons of the efficiencies of tested culture media, in terms of exclusive families of the uncultured phyla/divisions detected, plant-based culture media exclusively recovered 3 families belonging to BRC1, *Microgenomates* (OP11), and ZB3. Plant-based media also shared 3 families belonging to FBP, LCP, and *Parcubacteria* (OD1) with culture-independent communities (Fig. S4 and Data S1).

### Pairwise comparisons among culture media based on individual OTU abundance

We compared the abundance of individual OTUs in order to assess culture medium potentials to support the growth of naturally less abundant bacteria, *i.e.* all bacterial phyla, except for *Proteobacteria*, *Firmicutes*, and *Bacteroidetes*. In these analyses, we only considered OTUs that displayed significant differences in abundance between nutrient agar and plant-based culture media.

In the endorhizosphere, out of 1,747 different OTUs detected, 520 (29%) displayed significant differences in abundance between plant-based culture media and nutrient agar. Among these OTUs, 345 exhibited higher abundance on plant-based culture media samples than the 175 on nutrient agar (Fig. S5A). PCoA revealed a distinct separation between the communities of nutrient agar and plant-based culture media along PCoA-1 and PCoA-2, which explain variations of 56 and 23%, respectively (Fig. S5B). The abundance-based heatmap-annotated dendrogram of these significantly different OTUs, in total or when eliminating the three biggest phyla (*Proteobacteria*, *Firmicutes*, and *Bacteroidetes*), clearly demonstrated markedly higher abundance in plant-based culture media (Fig. S5C and D). We selected and clustered OTUs belonging to the unculturable candidate phyla/divisions (54 OTUs) for a closer examination (Fig. S5E and Data S2). We found that 46 of these OTUs were significantly enriched on plant-based culture media. They belonged to the following unculturable candidate divisions/phyla: *Parcubacteria* (OD1), *Microgenomates* (OP11), *Omnitrophica* (OP3), *Saccharibacteria* (TM7), *Latescibacteria* (WS3), *Dependentiae* (TM6), *Gracilibacteria* (GN02), *Modulibacteria* (KSB3), *Hydrogenedentes* (NKB19), *Cloacimonetes* (WWE1), *Atribacteria* (OP9), AC1, BRC1, FBP, GAL15, and LCP (Fig. S5E, the outer 3 rings). Similar results were observed for the ectorhizosphere. A total of 180 out of the 1,755 different OTUs (10%) showed significant differences in abundance between the culture media tested (Fig. S6), with 108 showing significantly higher abundance on plant-based media and the remainder on nutrient agar. PCoA and heatmap clustering displayed the clear separation of the abundance of these OTUs among the culture media tested (Fig. S6).

### Pairwise comparisons among culture media based on phylum abundance

**Fig. 4:**
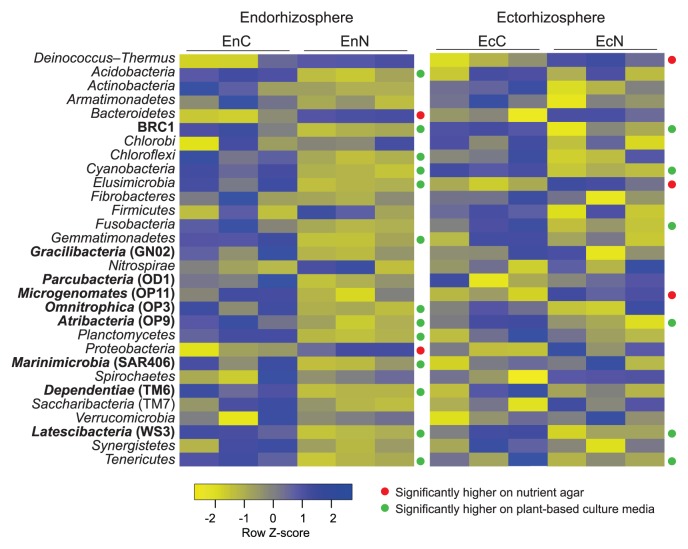
Heatmap representing significant differences between tested culture media (nutrient agar and plant-based culture media) in respect of the weighted abundance of 30 phyla, representing more than 95% of all detected OTUs, in maize compartments of the endorhizosphere and ectorhizosphere. The phyla indicated in bold are those lacking even a single representative isolate.

We employed the Student’s *t*-test to measure the significance of abundance at the phylum level in consideration of the sum of the hybridization scores of all OTUs of each phylum. In the endorhizosphere, nutrient agar only displayed significantly higher abundance (*P*≤0.05) in the two major phyla: *Proteobacteria* and *Bacteroidetes*. In contrast, plant-based culture media significantly enriched 13 phyla different from nutrient agar (Fig. 4). They included the following 6 not-yet-cultured candidate divisions/phyla: BRC1, *Omnitrophica* (OP3), *Atribacteria* (OP9), *Dependentiae* (TM6), *Latescibacteria* (WS3), and *Marinimicrobia* (SAR406), in addition to 7 fastidious not-easily-cultured phyla: *Acidobacteria*, *Chloroflexi*, *Cyanobacteria*, *Elusimicrobia*, *Gemmatimonadetes*, *Planctomycetes*, and *Tenericutes* (which includes “*Candidatus* Phytoplasma”) (Fig. 4). Of particular note was the recovery of the *Candidatus* Phytoplasma genus, a plant pathogen that is known to lack a single representative isolate; these were specifically enriched with higher abundance on the tested plant-based culture media for both root spheres (Data S3).

In the ectorhizosphere, differences among the tested culture media were confined to only 9 phyla; 6 were significantly enriched on plant-based culture media (BRC1, *Cyanobacteria*, *Atribacteria* [OP9], *Latescibacteria* [WS3], *Tenericutes*, and *Fusobacteria*) and 3 on nutrient agar (*Deinococcus-Thermus*, *Elusimicrobia*, and *Microgenomates* [OP11]) (Fig. 4).

## Discussion

Horizontal spatial divergence in the bacterial community structure across the soil-root system was attributed to proximity to the root core, and considered to be a key factor mediating variations within root-associated microbial communities (13, 25, 37). Therefore, the nutrient diversity and balance provided within the plant milieu drive natural selection towards a characteristic microbiome fingerprint that identifies a given plant host. In this respect, the selection/prediction of suitable culture media for the *in vitro* cultivation or *in situ* recovery of elements of the plant microbiome is problematic, even with recent databases that guide the selection of compatible culture media (45). We consider the use of culture media based on the nutritional make-up of the natural environment to be the proper approach for investigating the existing microbial population without discriminating against less abundant phyla. Accordingly, bringing the environment into the laboratory is a profitable approach and has already served as the basis for one of the most heralded success stories of bringing bacteria into culture (6, 16, 35, 54, 56, 59).

Plant materials were successfully introduced as the sole source of nutrients (plant-only-based culture media) for culturing rhizobacteria. These culture media have proved to be more advantageous in the cultivation of rhizobacteria than other standard artificial culture media (23, 43, 51, 62). One of the main reasons for the success of plant-based culture media to widen the scope of culturing not-yet-cultured rhizobacteria is their particular composition of C and N compounds, and their richness in metal ions, vitamins, and cofactors (51). These are the largest differentiators among defined growth conditions for strains within a given genus or family (45). This concept may be extended further to include other environmental interactions, such as quorum sensing molecules and siderophores in bacterial co-cultures that enable the growth of other unculturable organisms (56, 59).

Many previous studies that compared culture-dependent and -independent methods did not consider the effect of culture media—in terms of the origin, nature, and concentrations of nutrients—on the composition of bacterial communities (2, 28, 48, 49, 55). Therefore, we utilized plant-only-based culture media because of the complexity and diversity of nutrients delivered by plant materials. In the present study, we used the high-throughput “G3-PhyloChip Microarray” for the first time to characterize bacterial community compositions resolved by culture-dependent and -independent techniques. We extensively compared the incidence and abundance of the culturable bacterial communities of maize root compartments, with a focus on the effects of culture media and the range of not-yet-cultured candidate bacterial phyla/divisions and rarely-isolated phyla.

Consistent with our previous findings that were obtained based on a PCR-DGGE analysis (51), our PhyloChip assay confirmed higher taxa richness in plant-based culture media than in nutrient agar, more so for the endorhizosphere than for the ectorhizosphere. This result supports our theory that plant-based culture media create uniquely balanced and supportive environments that are more suitable for endophytes than ectophytes, and emphasize the strong imprint of the plant on its microbiome. This effect was partially observed when plant extracts were added as a supplement to standard culture media (14, 15, 41).

Comparisons of overall phylum abundance among the tested culture media clearly demonstrated the significant enrichment of plant-based culture media to not-yet-cultured candidate phyla/divisions: BRC1, *Omnitrophica* (OP3), *Atribacteria* (OP9), *Dependentiae* (TM6), *Latescibacteria* (WS3), and *Marinimicrobia* (SAR406), and other fastidious not easily-cultured phyla: *Acidobacteria*, *Chloroflexi*, *Cyanobacteria*, *Elusimicrobia*, *Gemmatimonadetes*, *Planctomycetes*, and *Tenericutes* (Fig. 4). The enrichment capacity of plant-based culture media was further demonstrated by the ratio of significantly enriched OTUs belonging to the not-yet-cultured candidate phyla/divisions (Fig. S5E). Of the 54 OTUs detected, most (46 OTUs) displayed higher abundance on plant-based culture media than on nutrient agar (8 OTUs), as represented by *Parcubacteria* (OD1), *Microgenomates* (OP11), *Omnitrophica* (OP3), *Latescibacteria* (WS3), *Dependentiae* (TM6), *Gracilibacteria* (GN02), *Modulibacteria* (KSB3), *Hydrogenedentes* (NKB19), *Cloacimonetes* (WWE1), *Atribacteria* (OP9), AC1, FBP, GAL15, BRC1, and LCP. Since the results of our DNA analysis targeted 10-d-old grown CFUs, the detection of unique taxa with the plant-based culture media used points mainly to the potential of these culture media to increase culturability, but does not appear to guarantee stable successive subculturing, *i.e.* temporal recovery. We experienced this particular situation while culturing the microbiota of the cactus “*Aloe arborescens*”; 40% of the μ-colonies that developed on homologous plant-based culture media failed to be successively subcultured (51, 62). In order to secure successive subcultures, more defined endophytic growth conditions, *e.g.* nutrient complexity, long-term incubation, adjustable gas phases, and/or co-culturing conditions, appear to be required.

Consistent with the findings reported by Stewart (56), we consider an “unculturable status” to not imply that an unculturable population “can never be cultured”, but rather to signify that we lack critical information on their biology and physiology. This obstructs our efforts in the *in vitro* culturing of these populations because of three principle mechanisms: very poor development in the form of overlooked μ-colonies, a change in state to “Viable But Not Culturable (VBNC)”, and the lack of endosymbiosis (Table S2).

Contrary to culture-independent methods that expose the wide diversity of the plant microbiome, most culture-dependent studies recover bacterial members belonging to the three main phyla (*Proteobacteria*, *Firmicutes*, and *Bacteroidetes*) (2, 6, 14, 28, 48). Therefore, we introduced plant-based culture media with the objective of recovering fastidious bacterial groups at the expense of fast-growing opportunistic bacteria. Plant-based culture media provide the culturable microbiome with diverse nutrients at concentrations that mimic the root milieu. Additionally, plant materials in the form of plant-based culture media (51, 62) or as a supplement to enrich standard artificial culture media (44) impaired the swarming motility of bacteria on agar plates, thereby halting their expansive progression and restricting the dominance of larger slimy colonies. In many cases, these slimy colonies masked the presence of some small colonies and/or μ-colonies. We noted that the natural make-up of nutrients in plant-based culture media resulted in the unique advantage of supporting the relatively slow-growers of confined μ-colonies (Fig. S2). Therefore, representatives of unculturable bacteria were reported to develop in the form of μ-colonies, *i.e.* successfully enriched but not visible to the naked eye (18, 21). These μ-colonies were the focus of several studies, in which fluorescence and on-chip microscopy were successfully used to facilitate the assessment of microbial abundance (30, 32). One of the highlights of these μ-colonies was the cultivation of the first representative isolate of the phylum TM7. After 20 years of trials, this candidate division was physiologically and genetically investigated, then successfully grown *in vitro* in the form of 20–200 μm μ-colonies and designated as “*Saccharibacteria*” (1, 12, 54). Since μ-colonies were detected among the culturable population of our tested plant-based culture media, the μ-colony phenomenon needs to be studied in more detail on plant-only-based culture media.

Some cells that appeared to lose viability during growth-arrested states were actually entering the “Viable But Not Culturable (VBNC)” states, which are characterized by the inability to form colonies under nutrient stress conditions, but with the continual maintenance of the proton motive force (PMF) (4, 8, 24). The widespread existence of these VBNC states in the environment and the lack of understanding of the triggers for recovering from these states have been proposed as one of the reasons why many bacteria remain uncultured in the laboratory (16). Thus, it is not attributed to the state of unculturability, but rather the inability to form a visible colony under routine standard cultivation conditions due to the growth-arrested state, low metabolic activity, and simultaneously increasing doubling time (4). Therefore, the higher abundance of many of these uncultured taxa on our tested plant-based culture media may be attributed to the activation of their growth and metabolic activity, leading to emergence from a growth-arrested state (4, 9). The use of diluted culture media together with an extension of the incubation period of up to three months successfully proved this theory (10, 27, 29, 57, 64). An example is the isolation of the first representative member of the candidate division OP10 “*Armatimonadetes*” as soon as the concentration of nutrients decreased to 1% of commonly used Trypticase Soy Agar (TSA) culture media. The secured isolate by itself resisted cultivation and subculturing on full-strength Luria-Bertani (LB) and TSA culture media (57).

Based on data derived from single-cell genomics as well as genome-resolved metagenomics, the majority of unculturable candidate divisions/phyla are of limited metabolic capacities because of their small-sized genomes (Table S2). Many of them are compelled to be symbionts with other higher organisms (1, 5, 26, 42).

An important example is *Candidatus* Phytoplasma spp., the genomes of which lack the basic genes responsible for the tricarboxylic acid cycle and the biosynthesis of sterols, fatty acids, and most amino acids (47). According to the ATCC and DSMZ culture collections, this genus does not have a single representative isolate. They are known to be plant pathogens and phloem symbiont/inhabitants, which allows them to achieve their metabolic needs. Due to their resolution with significant enrichment on our tested plant-based culture media, we consider the complexity and diversity of nutrients delivered through these plant-based culture media to fulfil their nutritional requirements, thereby mimicking the conditions that prevail in their natural habitat, the plant phloem (47). This was also confirmed by the successful *in vitro* recovery of this bacterium by constructing a complex culture medium to satisfy their nutritional requirements (7).

## Conclusions

Our results present conclusive evidence and strong support for the potential of using plant-based culture media to culture the plant microbiome, both for the purpose of increasing culturability and deciphering not-yet-cultured candidate phyla/divisions. In future studies, adopting novel methods associated with naturally-formulated culture media for further experiments is justified (23, 56). Rather than focusing on the populating subsets of organism-medium matrices to elucidate the key growth principles of these unculturables (45), these methods will help to improve the success rate for recovering as-yet-uncultivated divisions *in vitro* in order to investigate/foster their environmental impacts. We now have the ability to confidently predict that future advances with these methods will result in the target of tailored protocols and various strategies to increase culturability and isolate as many isolates as possible, known as “culturomics”, in plant microbiome studies.

## Supplementary methods
