# Supplementary material for: G3 PhyloChip Analysis Confirms the Promise of Plant-Based Culture Media for Unlocking the Composition and Diversity of the Maize Root Microbiome and for Recovering Unculturable Candidate Divisions/Phyla
Source: Microbes Environ. 2018 Sep 29;33(3):317–25. doi: 10.1264/jsme2.ME18023 (PMC6167109; doi:10.1264/jsme2.ME18023)
Supplement: Supplementary file 1 [file 33_317_s1.pdf]

Supplementary methods:

Amplified products were then concentrated using a solid-phase reversible immobilization method for the purification of PCR products and quantified by electrophoresis using an Agilent 2100 Bioanalyzer®. PhyloChip Control Mix™ was added to each amplified product. Bacterial 16S rRNA gene amplicons were fragmented, biotin labeled, and hybridized to the PhyloChip™ Array, version G3. PhyloChip arrays were washed, stained, and scanned using a GeneArray® scanner (Affymetrix). Each scan was captured using standard Affymetrix software (GeneChip® Microarray Analysis Suite). From each of the purified PCR products, 500 ng were fragmented and hybridized. Assuming an average GC content of 54% (based on Greengenes database of 16S rRNA genes) and an amplicon length of 1,465 bps, 3.3+E11 (330 billion) molecules were assayed from each sample (11).

Second Genome's PhyloChip processing software, Sinfonietta, was used to execute a multi-stage process (11). The first stage of pixel summarization of the florescent image and array scaling were conducted as previously described by Hazen *et al.* (5). Array fluorescence intensity (FI) of each pixel and image was collected as integer values ranging from 0 to 65,536 providing 216 distinct FI values.

The summary of FI for each single probe feature on the array was calculated by ranking the FI of the central 9 of 64 image pixels and using the value of 75th percentile. Background was defined separately for each G+C class as the median feature FI of all non-16S control probes for a given G+C class. Next, all probes on the array were scaled by multiplication with a single factor so that average FI of the probes perfectly matching the PhyloChipTM Control Mix of non-16S spikes were equal. FI values from redundant probes were averaged to generate the simple probe-level table representing the responses of 994,980 unique 25-mers across all samples. Pairs of probes are two probes with similar but non-identical sequences which align along ≥ 23 bases with ≥ 1 mismatch or gap as determined by blastn (2). (-word_size 8 -dust no -perc_identity 92 –e value 0.005 -penalty -1). Although all probes can produce minor fluorescence from non-specific hybridization, if a sequence-specific hybridization has occurred the probe complementing the target will be brighter than its mis-matching mate as has been observed in 70% of controlled experiments (6). As a general caution, perfect matching probes (PM) were considered positive if they fulfilled the

following criteria in comparison to their corresponding mis-matching probes (MM). A) (PM minus MM) > 3*(G+C class-specific background) AND (PM minus MM)/(PM plus MM) > 0.189.

Only PM FI from probes observed as positive in at least 4 experiments were exported from all experiments then rank-normalized in Sinfonietta software and used as input to empirical probe-set discovery. Probes were clustered into probe-sets based on both correlations in FI across all biological samples and taxonomic relatedness. Where multiple clustering solutions were available, higher correlation coefficients were favored over lower, taxonomic relatedness at the species level was favored over higher ranks, and sets composed of more probes were favored over less. All probe sets contained ≥ 5 probes. The empirical OTU (eOTU) tracked by a probe set was taxonomically annotated against the May 2013 release of Greengenes from the combination of the 8-mers contained in all probes of the set (7). The mean FI for each eOTU and each sample was calculated and then rank-normalized within each sample. These values are referred to as the hybridization score (HybScore) used in abundance-based analysis. The proportion of probes for an eOTU that are observed as positive in a sample is referred to as the positive fraction (pf). An eOTU was considered present in a sample where pf ≥ 0.8, and only eOTUs passing the pf cutoff are included in the AT and BT tables below.

Table S1. Adonis test and significance summary between OTUs of culture-dependent and independent microbial communities, characterized by G3-PhyloChip microarray analysis, based on weighted and unweighted UniFrac distance matrices. Significant differences ($P \leq 0.05$) are indicated by *.

column_name	Comparison_between	sample_counts	WUniFrac	UniFrac
Rootsphere	Ec | En	9 | 9	0.009*	0.016*
Milieu	C | N | R	6 | 6 | 6	0.001*	0.001*
comcategor	EcC | EcN | EcR | EnC | EnN | EnR	3 | 3 | 3 | 3 | 3 | 3	0.001*	0.001*
RvsC	C | R	6 | 6	0.003*	0.004*
RvsN	N | R	6 | 6	0.005*	0.002*
NvsC	C | N	6 | 6	0.023*	0.065

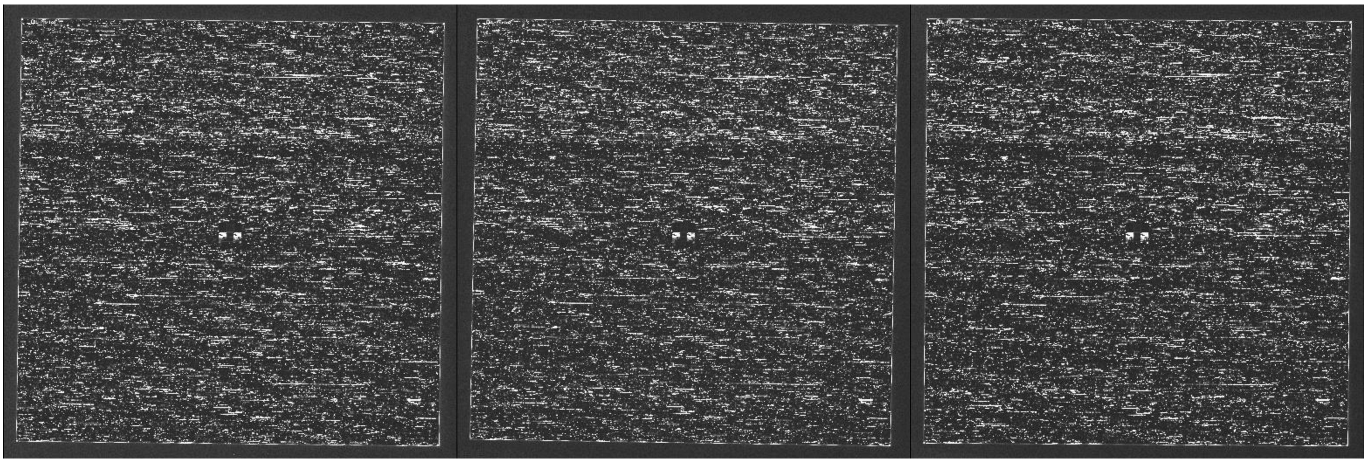

Fig. S1. Scan images showing the hybridization scores (HybScores) of the analyzed G3-PhyloChip microarrays used in the study and carried out by Second Genome Inc., San Francisco, CA, USA.

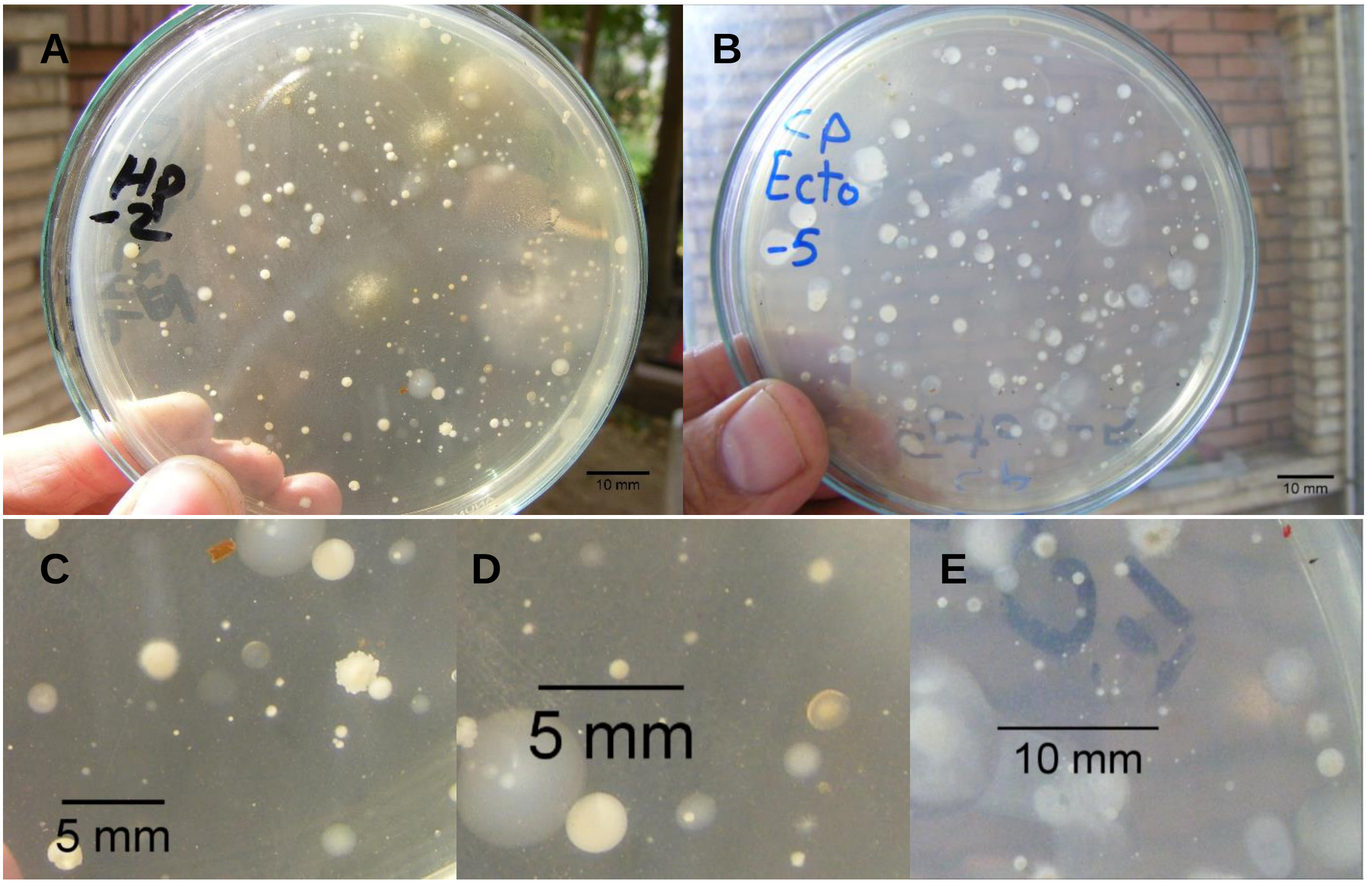

Fig. S2. Microcolonies developed on plant-only-based agar plates incubated for more than 10-days: A & B, overall predominance of microcolonies; C, D, & E, close-ups of microcolonies together with scale-bar to illustrate their microscale diameter.

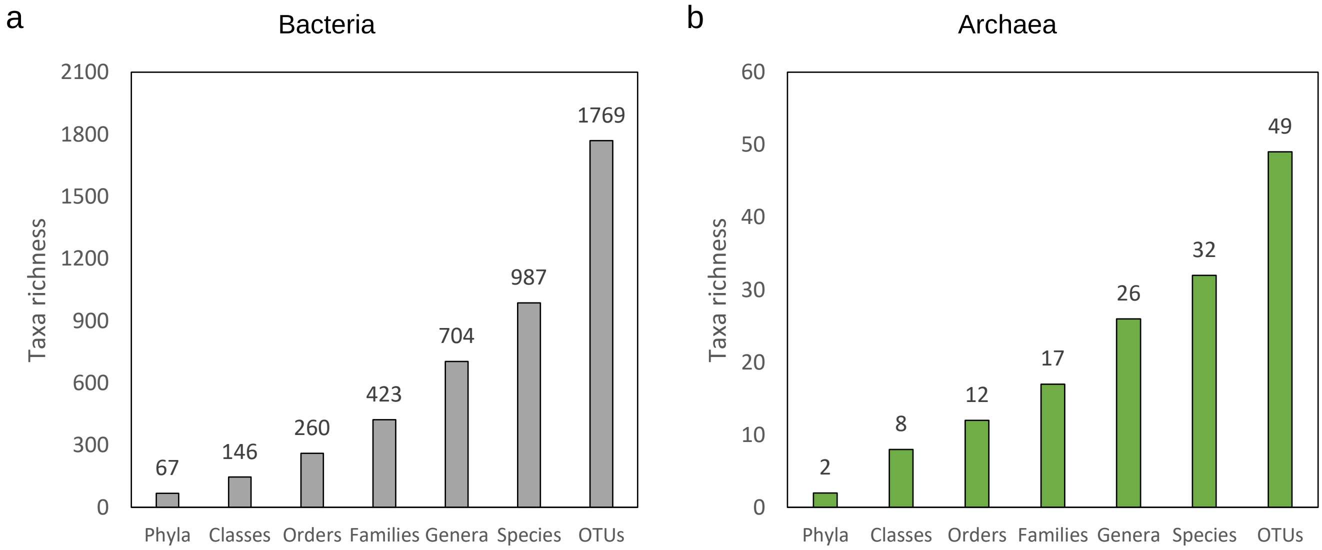

Fig. S3. Whole bacterial (a) and archaeal (b) richness of the maize root microbiome, detected using culture dependent and culture independent methods, at different taxonomic levels.

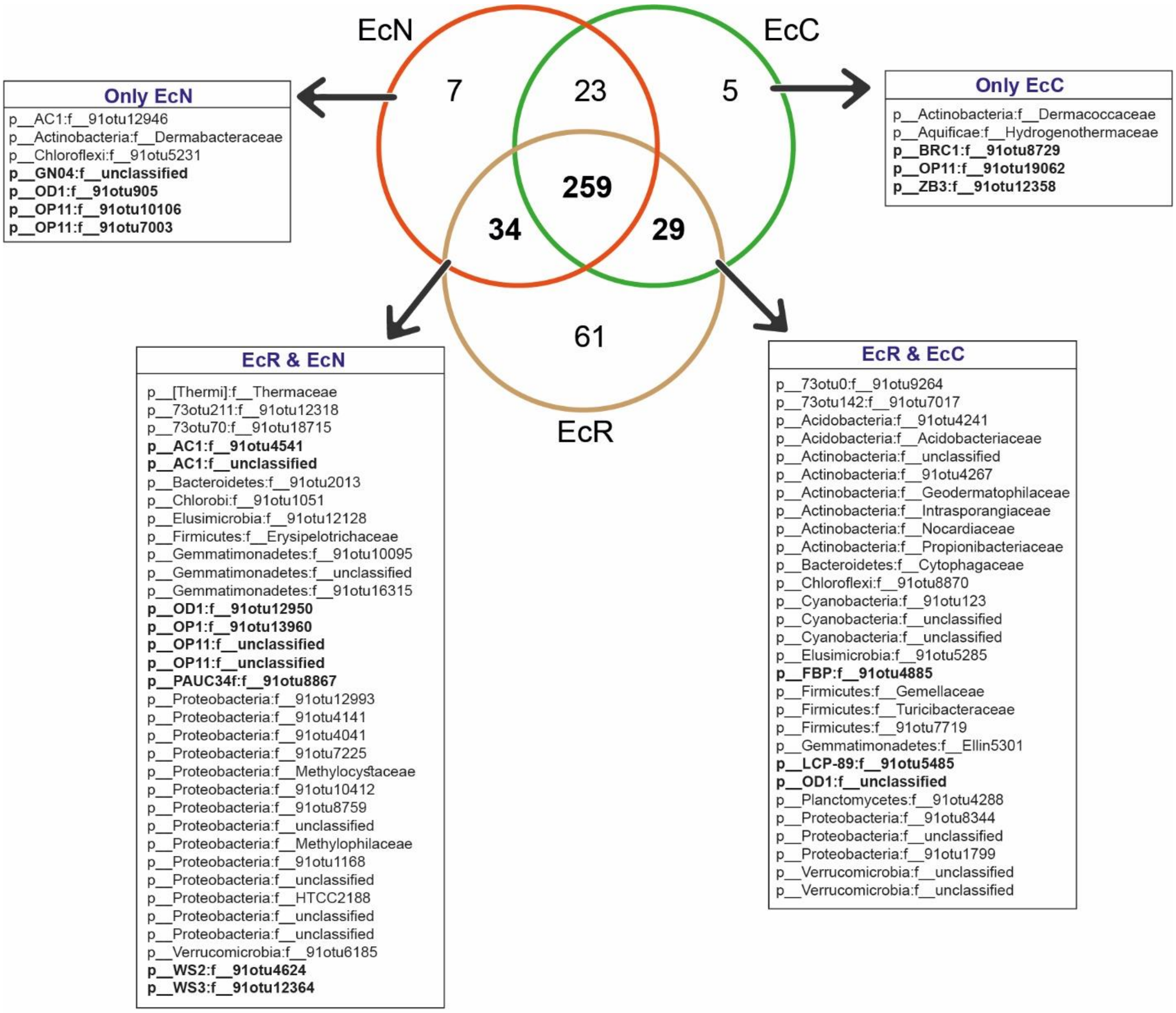

Fig. S4. Overlapping of culture-dependent (on plant-based culture medium and nutrient agar) and culture independent bacterial communities of maize root ectorhizosphere. Venn diagram at family level for bacterial communities displaying the unique and overlapping families; families exclusively grown on only one of our tested media are shown in the linked boxes, and not-yet-cultured candidate divisions are marked in bold. (**EcN**, nutrient agar; **EcC**, plant-based culture medium; **EcR**, maize ectorhizosphere).

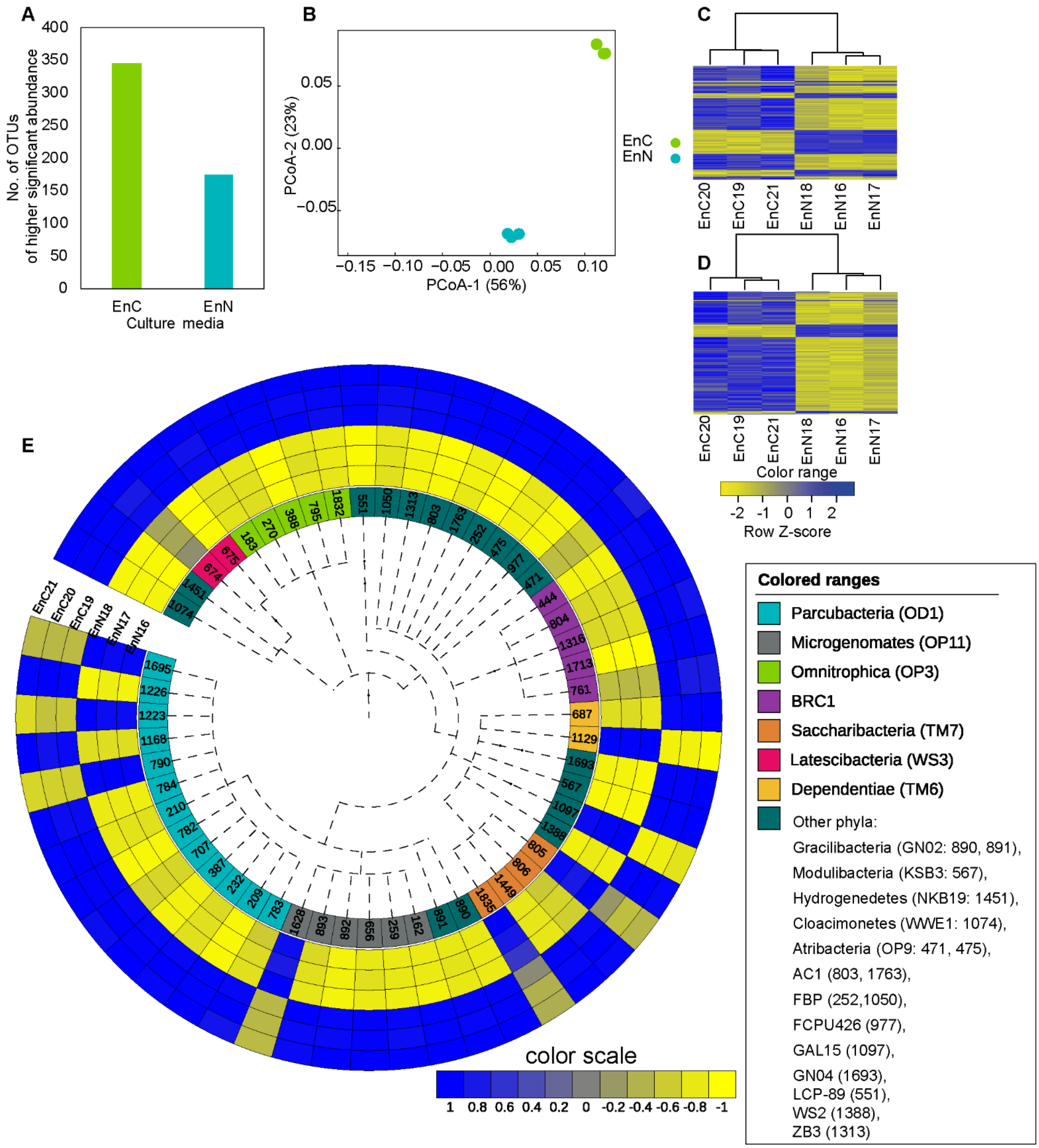

Fig. S5. The total 520 OTUs of the maize root Endorhizosphere (350 OTUs on plant-based medium and 170 OTUs on nutrient agar) that displayed significant differences in abundance among all of the three tested samples of nutrient agar (EnN16, EnN17 and EnN18) and the three tested samples of plant-based culture medium (EnC19, EnC20 and EnC21). **A**, bar-graph representing the absolute number of OTUs of significant higher abundance in either culture media; **B**, Principal Coordinates Analysis (PCoA); **C**, heatmap displaying the weighted abundance of the 520 OTUs; **D**, heatmap displaying the weighted abundance excluding the OTUs of the big-three phyla (*Proteobacteria*, *Firmicutes*, *Bacteroidetes*); **E**, Circular phylogenetic tree representing the 54 OTUs of uncultured bacterial phyla, annotated with heatmap of weighted abundance. Numbers of the inner ring refer to OTUs numbers present in the Data S1 (available upon request for data depository). Numbers between parentheses in “colored range” box indicate the OTU codes used in the circular dendrogram.

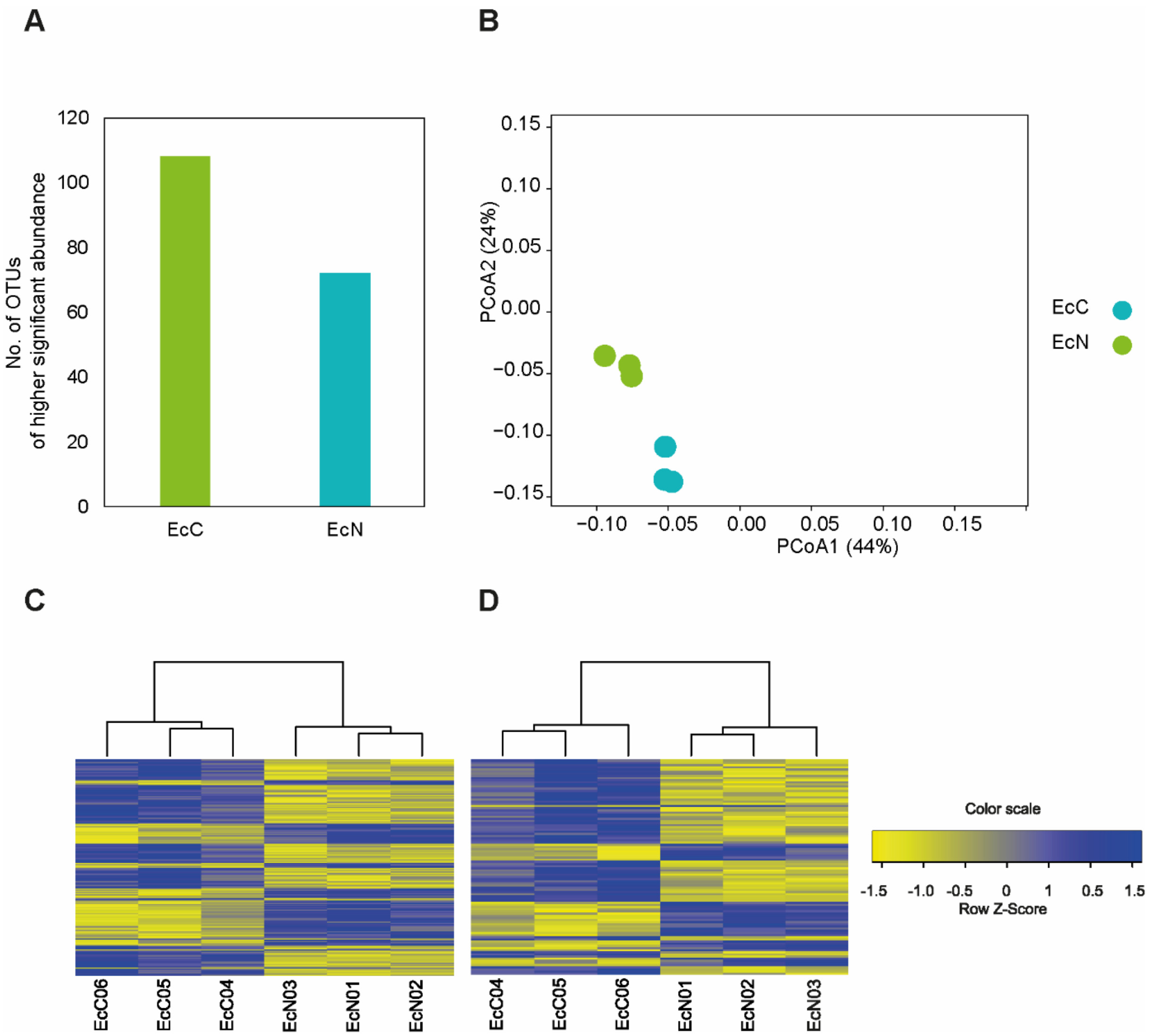

Fig. S6. The OTUs of the maize ectorhizosphere that displayed significant differences in abundance the three tested samples of nutrient agar (EcN01, EcN02 and EcN03) and the three tested samples of plant-based culture media (EcC04, EcC05 and EcC06). **A**, bar-graph representing the absolute number of OTUs of significant higher abundance; **B**, Principal Coordinates Analysis (PCoA); **C**, heatmap representing all OTUs of significant abundance; **D**, heatmap representing all OTUs of significant abundance excluding the big-three phyla (*Proteobacteria*, *Firmicutes*, *Bacteroidetes*).

Table S2. Candidate division/phyla particularly those enriched on plant-only-based culture media in this study, and previously reported in literature via metagenomics analyses among plant microbiomes. Related references are indicated in parentheses.

Candidate phyla/division	Updated taxonomic nomenclature	Number of OTUs reported in this study	Previous metagenomics reports on plant microbiomes	Available Genome-derived information regarding the recalcitrant culturability
AC1	NA	4	Cotton rhizosphere (12)	NA
BRC1	NA	12	Cotton rhizosphere (12)	NA
FBP	NA	3	Cotton rhizosphere (12)	NA
GN02	Gracilibacteria	9	Cotton rhizosphere (12)	NA
NKB19	Hydrogenedentes	1	Cotton rhizosphere (12)	NA
OD1	Parcubacteria	29	Cotton rhizosphere (12)	Gene sets for biosynthesis of cofactors, amino acids, nucleotides, and fatty acids are absent entirely or greatly reduced. They also lack some activities conserved in almost all other known bacterial genomes, including signal recognition particle, pseudouridine synthase A, and FAD synthase (8).
OP9	Atribacteria	5	Cotton rhizosphere (12)	Atribacteria' are likely to be heterotrophic anaerobes that lack respiratory capacity (9).
OP8	Aminicenantes	4	Cotton rhizosphere (12)	NA
OP3	Omnitrophica	10	Cotton rhizosphere (12)	NA
OP10	Armatimonadetes	11	Maize rhizosphere and several species of angiosperms (5, 10)	NA
OP11	Microgenomates	12	Cotton rhizosphere (12)	NA
TM6	Dependentiae		Cotton rhizosphere (12)	Lack complete biosynthetic pathways for various essential cellular building blocks including amino acids, lipids, and nucleotides. These and other features identified in the TM6 genomes such as a degenerated cell envelope, ATP/ADP translocases for parasitizing host ATP pools, and protein motifs to facilitate eukaryotic host interactions indicate that parasitism is widespread in this phylum (13).
TM7	Saccharibacteria	17	Maize rhizosphere (3, 10)	Absence of key genes for the Embden-Meyerhof (phosphofructokinase) and Entner-Doudoroff (KDPG aldolase) pathways suggest that TM7 can use only the pentose phosphate and heterolactic fermentation pathways for which all key genes were identified (1).
WPS-2	NA	3	several species of angiosperms (5)	NA
WS2	NA	3	Cotton rhizosphere (12)	NA
WS3	Latescibacteria	6	Rice root rhizoplane, rhizosphere, endosphere<br>maize rhizosphere (4, 10)	Assumed to have slow growth rate due to possession of a relatively large genome size and a single rRNA operon (14).
ZB3	Ignavibacteriae	2	Cotton rhizosphere (12)	NA

Supplementary References

1. Albertsen M., P. Hugenholtz, A. Skarshewski, K.L. Nielsen, G.W. Tyson, and P.H. Nielsen. 2013. Genome sequences of rare, uncultured bacteria obtained by differential coverage binning of multiple metagenomes. Nat. Biotechnol. 31:533–8

2. Altschul S.F., W. Gish, W. Miller, E.W. Myers, and D.J. Lipman. 1990. Basic local alignment search tool. J. Mol. Biol. 215:403–10

3. Bulgarelli D., M. Rott, K. Schlaeppi, et al. 2012. Revealing structure and assembly cues for Arabidopsis root-inhabiting bacterial microbiota. Nature. 488:91–5

4. Edwards J., C. Johnson, C. Santos-Medellín, E. Lurie, N.K. Podishetty, S. Bhatnagar, J.A. Eisen, and V. Sundaresan. 2015. Structure, variation, and assembly of the root-associated microbiomes of rice. Proc. Natl. Acad. Sci. U. S. A. 112:E911-20

5. Fitzpatrick C.R., J. Copeland, P.W. Wang, D.S. Guttman, P.M. Kotanen, and M.T.J. Johnson. 2018. Assembly and ecological function of the root microbiome across angiosperm plant species. Proc. Natl. Acad. Sci., p. 201717617

6. Furusawa C., N. Ono, S. Suzuki, T. Agata, H. Shimizu, and T. Yomo. 2009. Model-based analysis of non-specific binding for background correction of high-density oligonucleotide microarrays. Bioinformatics. 25:36–41

7. McDonald D., M.N. Price, J. Goodrich, E.P. Nawrocki, T.Z. DeSantis, A. Probst, G.L. Andersen, R. Knight, and P. Hugenholtz. 2012. An improved Greengenes taxonomy with explicit ranks for ecological and evolutionary analyses of bacteria and archaea. ISME J. 6:610–8

8. Nelson W.C., and J.C. Stegen. 2015. The reduced genomes of Parcubacteria (OD1) contain signatures of a symbiotic lifestyle. Front. Microbiol. 6:1–14

9. Nobu M.K., J.A. Dodsworth, S.K. Murugapiran, et al. 2016. Phylogeny and physiology of candidate phylum “Atribacteria” (OP9/JS1) inferred from cultivation-independent genomics. ISME J. 10:273–286

10. Peiffer J.A., A. Spor, O. Koren, Z. Jin, S.G. Tringe, J.L. Dangl, E.S. Buckler, and R.E. Ley. 2013. Diversity and heritability of the maize rhizosphere microbiome under field conditions. Proc. Natl. Acad. Sci. U. S. A. 110:6548–53

11. Probst A.J., P.Y. Lum, B. John, E.A. Dubinsky, Y.M. Piceno, L.M. Tom, G.L. Andersen, Z. He, and T.Z. de Santis. 2014. Microarray of 16S rRNA Gene Probes for Quantifying Population Differences Across Microbiome Samples. In *Microarrays Current Technology, Innovations and Applications Caister*, ed Z He. Caister Academic Press

12. Qiao Q., F. Wang, J. Zhang, Y. Chen, C. Zhang, G. Liu, H. Zhang, C. Ma, and J. Zhang. 2017. The Variation in the Rhizosphere Microbiome of Cotton with Soil Type, Genotype and Developmental Stage. Sci. Rep. 7:1–10

13. Yeoh Y.K., Y. Sekiguchi, D.H. Parks, and P. Hugenholtz. 2015. Comparative genomics of candidate phylum TM6 suggests that parasitism is widespread and ancestral in this lineage. Mol. Biol. Evol. 33:915–927

14. Youssef N.H., I.F. Farag, C. Rinke, S.J. Hallam, T. Woyke, and M.S. Elshahed. 2015. In Silico Analysis of the Metabolic Potential and Niche Specialization of Candidate Phylum “Latescibacteria” (WS3). PLoS One. 10:e0127499
